# Safety of transesophageal echocardiography during transcatheter edge-to-edge tricuspid valve repair: A single-center experience

**DOI:** 10.3389/fcvm.2022.856028

**Published:** 2022-10-11

**Authors:** Katharina Hellhammer, Robert Schueler, Mareike Eißmann, Brigitte Schumacher, Alexander Wolf, Oliver Bruder, Thomas Schmitz, Moritz Lambers

**Affiliations:** ^1^Contilia Heart and Vascular Center, Department of Cardiology and Angiology, Elisabeth Hospital Essen, Essen, Germany; ^2^Department of Internal Medicine and Gastroenterology, Elisabeth Hospital Essen, Essen, Germany; ^3^Faculty of Medicine, University Duisburg-Essen, Essen, Germany; ^4^Faculty of Medicine, Ruhr University Bochum, Bochum, Germany

**Keywords:** transcatheter tricuspid valve repair, interventional imaging, transesophageal echocardiography, safety, complications

## Abstract

**Objectives:**

We aimed to determine transesophageal echocardiography (TEE) related complications during Transcatheter edge-to-edge tricuspid valve repair (TTVR).

**Background:**

Transesophageal echocardiography is essential to guide structural heart disease (SHD) interventions. TTVR has become an evolving procedure for high-risk patients not suitable for surgery. Whether this complex procedure is associated with TEE related complications is not known so far.

**Methods:**

We retrospectively analyzed 64 consecutive patients undergoing TTVR between 2019 and 2021 with the TriClip system (Abbott, Chicago, IL, USA) at our center. All procedures were performed under general anesthesia (GA). TEE related complications were classified as major and minor complications.

**Results:**

Transesophageal echocardiography related complications were observed in two patients (3.1%) with one major complication (1.6%) and one minor complication (1.6%). In one patient perforation of the esophageal mucosa requiring red blood cell transfusion was observed, the other patient had hematemesis due to minor esophageal and gastric lesions without the need for blood transfusion. Both patients recovered during hospital stay with no persistent symptoms at discharge.

**Conclusions:**

Transesophageal echocardiography related complications during TTVR are clinically relevant occurring in 3.1% of the patients. Further investigations are needed to identify potential risk factors and patients at high risk to develop a TEE related complication in the course of TTVR.

## Introduction

Transcatheter structural heart disease (SHD) interventions for left sided pathologies have become an inherent part in daily clinical practice. For tricuspid regurgitation (TR) treatment options have been limited so far. While tricuspid repair during left heart surgery is widely accepted in patients with severe primary or secondary TR (1), isolated repair of the tricuspid valve has shown to be unfavorable due to high surgical risk (2, 3). As TR is a highly prevalent disease, associated with high morbidity and mortality (4, 5), the clinical need for treatment options remains essential. Recently, transcatheter edge-to-edge tricuspid valve repair (TTVR) with the TriClip system (Abbott, Chicago, IL, USA) has emerged as a safe and (6) effective treatment option as current data suggests (7, 8). Transesophageal echocardiography (TEE) is indispensable to guide this complex intervention. Of note, deep transesophageal and transgastric views are necessary to evaluate clip position, grasping of the leaflets and leaflet insertion. Complications which have been described to be associated with periinterventional TEE are bleeding events of the upper gastro-intestinal tract, perforations of the esophagus or esophagogastric tears, dysphagia or lesions of the oropharynx (6, 9, 10). A complication rate ranging from 0.2 to 1.2% has been reported, considering these data were raised from patients undergoing cardiac surgery (6, 11). Recent data focusing on TEE related adverse events during transcatheter structural heart interventions reported a higher event rate of 5.3%−6.1% (10, 12). Patients undergoing TTVR were not included in these studies.

Whether the manipulation of the TEE probe during this complex procedure is associated with adverse events is not known so far. We therefore aimed to evaluate the incidence of TEE related complications in patients undergoing TTVR.

## Methods

In a single-center study we analyzed 64 consecutive patients undergoing TTVR with the TriClip system between 04/2019 and 06/2021. All patients referred to this procedure were included in this study, no exclusion criteria were defined. All interventions were performed under general anesthesia (GA). Procedural steps have been described previously (7). In brief, the procedural steps are as follows: after obtaining a femoral vein access, the guide is positioned in the junction of the inferior vena cava and right atrium. Fluoroscopy and 2/3D transesophageal echocardiography is used to guide the procedurals steps. The TriClip delivery system is placed over the tricuspid valve and properly orientated perpendicular to the line of coaptation of the tricuspid valve leaflets. The clip is opened and advanced into the right ventricle. After optimal positioning, the clip is pulled back and the leaflets are grasped. Leaflet insertion, reduction of TR and orientation of the clip is evaluated. In case of insufficient reduction of TR or inadequate leaflet insertion, the clip can be re-opened to perform another grasping maneuvre.

Baseline patient characteristics, procedural data and in-hospital data were obtained. Laboratory results were taken 1 day prior to the intervention. Oral anticoagulation (NOAK) was paused 1 day prior to procedure. In case of oral anticoagulation with a vitamin k antagonist, an international normalized ration (INR) ≤ 2 was obtained before the patient was referred to the intervention. Procedure time was defined as time from start of the procedure till final closure of femoral access site. Time under TEE was defined as time starting from insertion of the probe till retraction of the probe at the end of the procedure. TEE was conducted using a GE Vivid™ E95 with a 6VT-D probe (GE Healthcare Systems, USA) and was performed by a cardiologist with wide experience in transesophageal echocardiography and guidance of transcatheter structural heart interventions. Transcatheter tricuspid valve repair was performed by two experienced interventional cardiologists, performing structural heart interventions for several years. An activated clotting time >250 s was obtained throughout the intervention by administration of unfractionated heparin. Procedural success was defined as implantation of at least one TriClip device with TR reduction of at least one grade. Tricuspid regurgitation was assessed using standard 2D color Doppler methods and graded using a five-class grading scheme: mild, moderate, severe, massive and torrential (13).

TEE related complications were classified as minor and major complications. A major complication was considered to meet one of the following criteria: (1) upper gastro-intestinal bleeding requiring inotropes or blood transfusion, (2) mechanical lesions as perforations of esophagus or stomach requiring endoscopic or surgical intervention, and (3) persistent dysphagia requiring further diagnostic. Minor TEE related complications were considered as dysphagia or perioral hypesthesia persisting within 24 h after procedure, hematemesis without need for blood transfusion.

Statistical analysis was performed using SPSS Statistic (IBM, Inc.). Procedure time, Time under TEE and device time were expressed as median (min-max) ± standard deviation. All other continuous variables were expressed as means ± standard deviation. Categorial variables were specified in percentage and absolute numbers.

## Results

**Figure 1:**
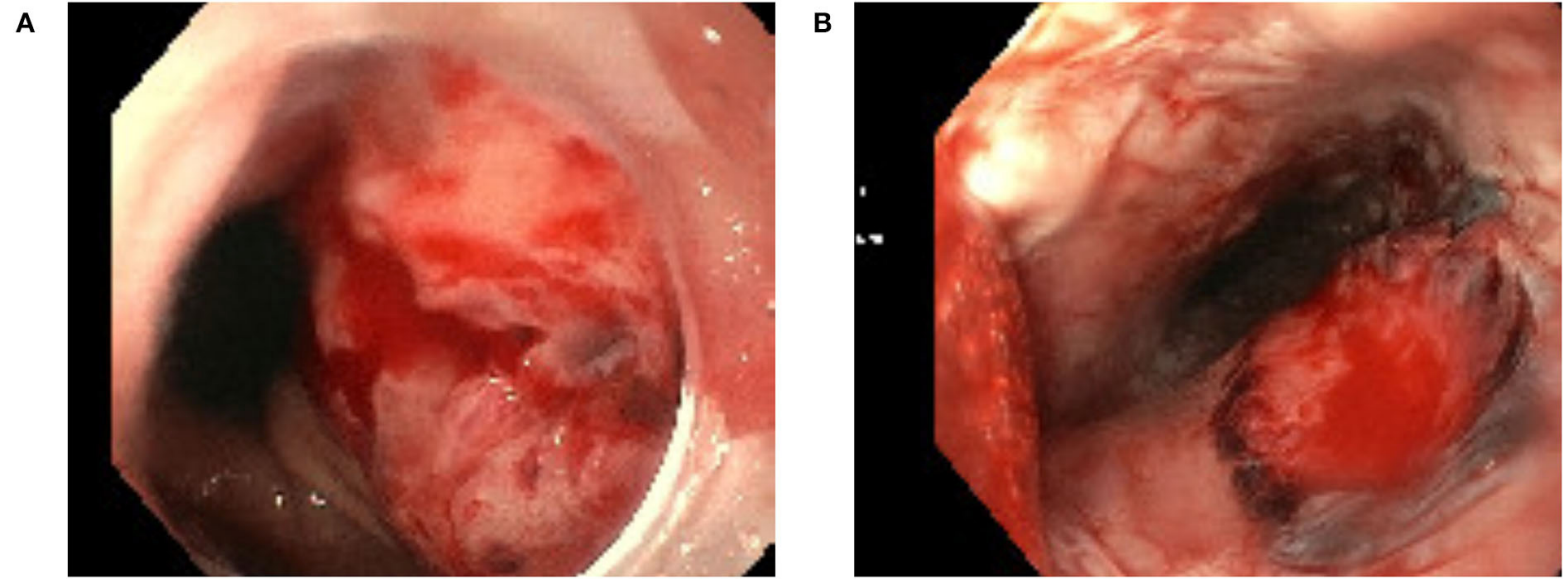
Esophageal mucosa perforation related to transesophageal echocardiography **(A)** and esophageal lesion related to transesophageal echocardiography **(B)**.

We observed a TEE related complication in 2 (3.1%) patients. Of these, one presented with a major complication due to a perforation of esophageal mucosa leading to an upper-gastro-intestinal bleeding which required transfusion of two red blood cell concentrates (Figure 1A). In the second patient, hematemesis occurred caused by multiple esophageal and gastric lesions as seen by endoscopy (Figure 1B). Hematemesis stopped spontaneously and no other actions had to be taken in this patient.

Transesophageal echocardiography related complications are listed in Table 1.

**Table 1:** TEE related complications.

	**All patients** **(*n* = 64)**
Overall complication rate, *n* (%)	2 (3.1)
Major complication, *n* (%)	1 (1.6)
Minor complication, *n* (%)	1 (1.6)

Mean age of our cohort was 80 ± 6.4 years. Patients presented with multiple comorbidites of which atrial fibrillation was the most common in 89.1% (*n* = 57) of the patients. Mean logistic EuroSCORE was 19.3 ± 13.0. Baseline characteristics are shown in Table 2.

**Table 2:** Baseline characteristics.

	**All patients** **(*n* = 64)**	**Patients with a TEE related complication** **(*n* = 2)**
Age, years	80 ± 6.2	81 ± 2.8
Male, *n* (%)	26 (40.6)	1 (50.0)
Body mass index (kg/m^2^)	24.9 ± 5.4	27.4 ± 6.6
Logistic EuroSCORE (%)	19.3 ± 13.0	15.2 ± 13.7
CHA_2_DS_2_-VASc Score	4.7 ± 1.2	4.5 ± 0.7
HAS-BLED Score	3.0 ± 0.9	2.5 ± 0.7
Coronary artery disease, *n* (%)	34 (53.1)	1 (50.0)
Previous heart surgery, *n* (%)	16 (25.0)	1 (50.0)
Atrial fibrillation, *n* (%)	57 (89.1)	2 (100)
pAVK, *n* (%)	5 (7.8)	0 (0.0)
cAVK, *n* (%)	7 (10.9)	0 (0.0)
OSAS, *n* (%)	3 (4.7)	0 (0.0)
COPD, *n* (%)	5 (7.8)	0 (0.0)
Diabetes mellitus, *n* (%)	13 (20.3)	0 (0.0)
Dialysis, *n* (%)	0 (0.0)	0 (0.0)
Anemia, *n* (%)	23 (35.9)	1 (50.0)
Immunotherapy, *n* (%)	1 (2.2)	0 (0.0)
Glomerular filtration rate (ml/min)	52 ± 19.2	63 ± 15.5
Hemoglobin (g/dl)	11.3 ± 1.7	10.8 ± 1.6
Thrombocytes, 103/μl	221 ± 77	218 ± 12.7
INR	1.3 ± 0.3	1.3 ± 0.1
Gastroesophageal pathologies, *n* (%)	7 (10.9)	1 (50.0)
-Gastroesophageal reflux disease, *n* (%)	3 (4.7)	1 (50.0)
-Chronic gastritis, *n* (%)	4 (6.3)	0 (0.0)
Dual antiplatelet therapy, *n* (%)	2 (3.1)	0 (0.0)
Oral anticoagulation, *n* (%)	55 (85.9)	2 (100)
Proton-pump inhibitor, *n* (%)	28 (43.8)	1 (50.0)

cAVK, cerebrovascular artery disease; COPD, chronic obstructive pulmonary disease; INR, international normalized ratio; OSAS, obstructive sleep-apnoe syndrome; pAVK, peripheral artery vascular disease; TEE, transesophageal echocardiography.

All patients underwent TTVR under GA. Median procedure time was 128 (46–220) min ± 36.7 with a successful procedure in 93.8% (*n* = 60) of the patients. In four patients the procedure was interrupted as the Clip could not be implanted due to insufficient grasping. No in-hospital deaths were observed. Procedural and in-hospital data is shown in Table 3.

**Table 3:** Procedural data and in-hospital data.

	**Patients without a TEE related complication** **(*n* = 62)**	**Patients with a TEE related complication** **(*n* = 2)**
Procedure time (min)	120 (46–220) ± 35.3	170 (149–190) ± 29.0
Time under TEE (min)	76 (22–180) ± 35.7	125 (99–150) ± 36.1
Number of implanted Clips	1.6 ± 0.7	1.5 ± 0.7
TR reduction (grades)	2.1 ± 0.8	2.0 ± 1.4
Total length of stay (day)	7.5 (3–23) ± 4.4	15 (9–21) ± 8.5
Length of stay on ICU (day)	0 (0–2) ± 0.4	3.5 (2–5) ± 2.1
Procedural success, *n* (%)	57 (91.9)	2 (100)
In-hospital mortality, *n* (%)	0 (0.0)	0 (0.0)

ICU, intensive care unit; TEE, transesophageal echocardiography; TR, tricuspid regurgitation.

We looked in detail at the two patients who presented with a TEE related complication (Tables 2, 3). Procedure time was prolonged with 170 (149–190) min ± 29.0. One patient was known to suffer from gastroesophageal reflux disease whereas in the second patient there was no history of previous gastroesophageal disease.

## Discussion

To our best knowledge, this is the first study evaluating the incidence of TEE related complications during TTVR. We observed TEE related complications in 3.1% of the patients.

Transcatheter edge-to-edge repair has recently successfully applied in patients with severe TR and shown to be safe and effective (7, 8, 14, 15). For procedural success guidance of TEE during this complex intervention is essential. Mid esophageal and transgastric views of the tricuspid valve are very important views while performing this procedure. As the intervention can be challenging with a prolonged procedure time, prolonged manipulation with the TEE probe may cause mechanical or bleeding complications. Complication rate of TEE in diagnostic routine has been described with 0.2%−0.5% (16). During SHD interventions, recent data found a TEE related adverse event in 6.1% of patients undergoing either percutaneous mitral valve repair (PMVR), closure of left atrial appendage or closure of paravalvular leakage (10). A prolonged procedural time under TEE was identified as a predictor for a TEE related complication and was highest in patients undergoing PMVR. These patients had a 10-fold higher risk to develop a major TEE related complication. In our study, the overall incidence of a TEE related complication was 3.1% with one major and one minor complication. We could not identify potential risk factors for the occurrence of a TEE related complication. The two patients presenting with a TEE related complication had a prolonged procedure time and therefore a longer time under TEE. Time under TEE may have an impact on adverse events as well in patients undergoing TTVR which has to be investigated in further studies. As transgastric views, where the probe is anteflexed toward the gastric fundus, are frequently used during this procedure, gastric lesions may occur more often, as observed in one of our patients.

Further considerations regarding the occurrence of TEE related complications may concern patient characteristics. Patients referred to TTVR are often a high-risk population presenting with multiple comorbidities (14) of which some may increase the risk of developing a TEE related complication. Besides, the procedure itself might as well be a risk factor for the occurrence of complications with its prolonged procedure time and therefore prolonged time under TEE. Previously reported complications after TTVR have been major bleedings, new onset of atrial fibrillation or single leaflet device attachment (14). These complications were not specific related to TEE. In our study, one of the two patients with a complication was suffering from gastroesophageal reflux disease. No routinely esophagogastroscopy was performed prior to intervention in our patients. This might be considered, at least in patients with known esophageal or gastric pathologies to decrease the risk for a TEE related complication. This complementary information might help to assess the periinterventional risk and identify patients who might need further treatment prior to intervention as for example high-dose PPI therapy.

## Conclusions

In conclusion, a TEE related was observed in 3.1% of the patients undergoing TTVR. Further prospective studies are needed to identify potential risk factors associated to the occurrence of a TEE related complications to improve the safety of TEE during TTVR.

## Limitations

The study has several limitations. First, it was a retrospective single-center study. Some events might not be recorded as they were self-limiting or felt not to be worth of mentioning in the medical data sheet. The number of complications might therefore be underestimated. As there was no systematic investigation of the upper gastrointestinal tract, subclinical lesions might as well be missed. Secondly, operator specific occurrence of events cannot fully be excluded, though procedures were performed by a fixed team, highly experienced in structural heart disease interventions. Thirdly, due to the limited number of events, no potential risk factors associated with TEE related complications could identified. This aspect has to be investigated in further prospective studies. Fourthly, the sample size was rather small to detect potential complications. Further multicentre, prospective studies are needed to obtain data from a large patient cohort and detect potential complications.

## Data availability statement

The raw data supporting the conclusions of this article will be made available by the authors, without undue reservation.

## Ethics statement

Ethical approval was not provided for this study on human participants because in accordance with the Local Ethics Committee requirements an ethical approval was not needed due to retrospective data analysis. Written informed consent for participation was not required for this study in accordance with the national legislation and the institutional requirements.

## Author contributions

KH and ML study concept, design, and supervision. KH, RS, ME, ML, BS, and AW acquisition and analysis or interpretation of data. KH writing and original draft preparation of the manuscript. TS, OB, and RS discussion and critical revision of the interpretation for important intellectual content. All authors reviewed the manuscript.

## Conflict of interest

The authors declare that the research was conducted in the absence of any commercial or financial relationships that could be construed as a potential conflict of interest.

## Publisher's note

All claims expressed in this article are solely those of the authors and do not necessarily represent those of their affiliated organizations, or those of the publisher, the editors and the reviewers. Any product that may be evaluated in this article, or claim that may be made by its manufacturer, is not guaranteed or endorsed by the publisher.

## Abbreviations

cAVK: cerebrovascular artery disease
COPD: chronic obstructive pulmonary disease
GA: general anesthesia
INR: international normalized ratio
OSAS: obstructive sleep-apnoe syndrome
pAVK: peripheral artery vascular disease
PMVR: percutaneous mitral valve repair
SHD: structural heart disease
TEE: transesophageal echocardiography
TR: tricuspid regurgitation
TTVR: transcatheter edge-to-edge tricuspid valve repair.
